# Production of Food and Feed Additives From Non-food-competing Feedstocks: Valorizing *N*-acetylmuramic Acid for Amino Acid and Carotenoid Fermentation With *Corynebacterium glutamicum*

**DOI:** 10.3389/fmicb.2018.02046

**Published:** 2018-09-24

**Authors:** Elvira Sgobba, Luisa Blöbaum, Volker F. Wendisch

**Affiliations:** Chair of Genetics of Prokaryotes, Faculty of Biology and CeBiTec, Bielefeld University, Bielefeld, Germany

**Keywords:** L-lysine, diamino pentane, lycopene, L-glutamate, biorefinery, food additives, peptidoglycan, *N*-acetyl-muraminic acid

## Abstract

*Corynebacterium glutamicum* is used for the million-ton-scale production of food and feed amino acids such as L-glutamate and L-lysine and has been engineered for production of carotenoids such as lycopene. These fermentation processes are based on sugars present in molasses and starch hydrolysates. Due to competing uses of starch and sugars in human nutrition, this bacterium has been engineered for utilization of alternative feedstocks, for example, pentose sugars present in lignocellulosic and hexosamines such as glucosamine (GlcN) and *N*-acetyl-D-glucosamine (GlcNAc). This study describes strain engineering and fermentation using *N*-acetyl-D-muramic acid (MurNAc) as non-food-competing feedstock. To this end, the genes encoding the MurNAc-specific PTS subunits MurP and Crr and the etherase MurQ from *Escherichia coli* K-12 were expressed in *C. glutamicum*Δ*nanR*. While MurP and MurQ were required to allow growth of *C. glutamicum*Δ*nanR* with MurNAc, heterologous Crr was not, but it increased the growth rate in MurNAc minimal medium from 0.15 h^-1^ to 0.20 h^-1^. When in addition to *murP-murQ-crr* the GlcNAc-specific PTS gene *nagE* from *C. glycinophilum* was expressed in *C. glutamicum*Δ*nanR*, the resulting strain could utilize blends of GlcNAc and MurNAc. Fermentative production of the amino acids L-glutamate and L-lysine, the carotenoid lycopene, and the L-lysine derived chemicals 1,5-diaminopentane and L-pipecolic acid either from MurNAc alone or from MurNAc-GlcNAc blends was shown. MurNAc and GlcNAc are the major components of the bacterial cell wall and bacterial biomass is an underutilized side product of large-scale bacterial production of organic acids, amino acids or enzymes. The proof-of-concept for valorization of MurNAc reached here has potential for biorefinery applications to convert non-food-competing feedstocks or side-streams to valuable products such as food and feed additives.

## Introduction

*Corynebacterium glutamicum* is a predominantly aerobic, rod-shaped, Gram-positive soil bacterium which is generally recognized as safe (GRAS). Since the 1960s, *C. glutamicum* was first used for the production of the flavor enhancer (Kinoshita et al., 1957) under biotin limiting conditions (Shiio et al., 1962). *C. glutamicum* was developed into an important organism for the biotechnological industry, producing amino acids on a million-ton scale (Wendisch, 2014). *C. glutamicum* has also been engineered to produce diamines, organic acids, carotenoids, proteins and biopolymers (Wendisch et al., 2016). Recently, metabolic engineering of *C. glutamicum* to expand its substrate scope allowed to use alternative carbon sources that do not have competing uses in the food industry (Zahoor et al., 2012). Access to the hexosamines GlcN (Uhde et al., 2013) and GlcNAc (Matano et al., 2014) has been reported, but utilization of the hexosamine MurNAc as alternative carbon source by *C. glutamicum* has not been described (Dominguez et al., 1997; Cramer and Eikmanns, 2007).

GlcN and GlcNAc can be gained by hydrolysis of chitin and chitosan that make up the arthropod exoskeleton and are present in fungal cell walls. Every year, circa 100 billion tons of chitin are produced in Nature and GlcNAc and GlcN can be obtained by acid hydrolysis (Chen et al., 2010; Zhang and Yan, 2017) and are available, e.g., from shrimp shell waste, an abundant side stream of the fishery industry. MurNAc and GlcNAc are the hexosamine constituents of peptidoglycan which makes up about 5% of the cell mass of Gram-negative bacteria and up to 20% of the cell mass of Gram-positive bacteria (Munoz et al., 1967; Reith and Mayer, 2011). The peptidoglycan constituents that can be found in all bacterial habitats have been used as indicators of bacterial biomass content in soils (Domsch, 1982). The Gram-positive *C. glutamicum* is the main producing organism for the annual production of 5 million tons of amino acids (Wendisch et al., 2016). Under the assumptions that (a) the same amount of cell dry weight is produced, (b) 20% of the cell dry weight is peptidoglycan and (c) about half of peptidoglycan is GlcNAc and MurNAc, about 500.000 tons of MurNAc and GlcNAc would be available from the amino acid fermentation industry. Biotechnological processes with bacterial hosts are used at the million-ton scale to produce secreted compounds such as organic acids, amino acids and enzymes. The spent biomass may be used in waste-to-energy applications either by thermal (e.g., incineration), thermo-chemical (e.g., torrefaction) or by biochemical treatments (e.g., anaerobic digestion). However, it is desirable to make use of the carbon and nitrogen containing hexosamine fraction of peptidoglycan in food and feed fermentation processes. The hexosamines may function both as carbon and nitrogen source for bacterial fermentations.

**FIGURE 1:**
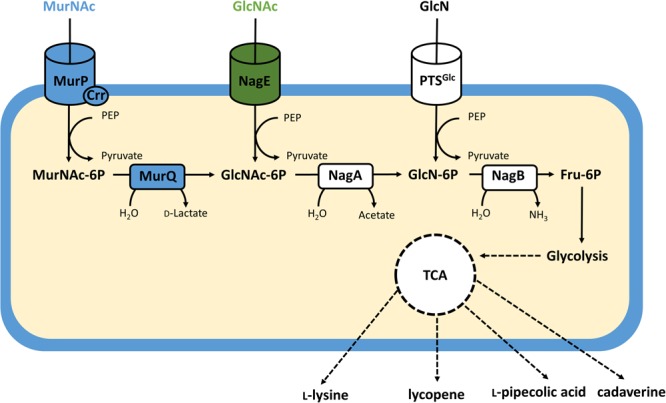
Schematic representation of the amino sugar catabolic pathways in recombinant *C. glutamicum*. Reactions from *E. coli* are given in blue, those from *C. glycinophilum* in green and native reactions are given in white with cylinders representing uptake systems and boxes representing enzyme reactions. Bold lines indicate individual reactions, dashed lines indicate multi-reaction conversions. MurP, MurNAc specific EIIBC PTS component; Crr, glucose-family specific PTS EIIA component; NagE, GlcNAc specific EIICBA PTS component; NagA, GlcNAc -6-phosphate deacetylase; NagB, GlcN-6-phosphate deaminase.

In chemical hydrolysis, the hexosamine fraction of peptidoglycan is accessible via enzymes of bacterial cell wall recycling. The degradation of the own cell wall by autolytic enzymes as a part of ll recycling is a common pathway in bacteria. When an *Escherichia coli lys dap* mutant was labeled with [^3^H]diaminopimelate for two generations and then chased, about 45% of its cell wall peptidoglycan was recycled per generation (Goodell and Schwarz, 1985; Uehara et al., 2006; Uehara and Park, 2008). The Gram-positive *Bacillus subtilis* can degrade, uptake and metabolize the cell wall component MurNAc in the stationary phase (Borisova et al., 2016). While all bacteria require cell wall peptidoglycan remodeling during growth and cell division, not all can utilize the monomeric components as carbon or nitrogen sources for growth. *C. glutamicum* may possess a minimal set of autolytic enzymes, however, many orthologs of the peptidoglycan degradation machinery from *E. coli* are absent (Dahl et al., 2004; Reith and Mayer, 2011). Catabolism of MurNAc in *E. coli* involves uptake and phosphorylation of MurNAc and GlcNAc via the phosphoenolpyruvate dependent phosphotransferase system (PTS). The MurNAc-specific PTS subunits are MurP and Crr (**Figure 1**). MurP, a two-domain protein that lacks a PTS-EIIA domain, is phosphorylated by EIIA^Glc^, a kinase encoded by the *crr* gene (carbohydrate repression resistance), which interacts with several members of the glucose PTS family (Nuoffer et al., 1988; Tchieu et al., 2001; Dahl et al., 2004). MurNAc-6-phosphate is further catabolized by the etherase MurQ (**Figure 1**) that cleaves the lactyl ether bond yielding GlcNAc-6-phosphate and D-lactate (Jaeger et al., 2005; Hadi et al., 2008). GlcNAc and GlcN are also taken up via the PTS with the specific subunits NagE (Plumbridge, 2009) and PTS^Man^ (Curtis and Epstein, 1975). NagA deacetylates GlcNAc-6-phosphate to GlcN-6-phosphate which is deaminated by NagB to the glycolytic intermediate fructose-6-phosphate (**Figure 1**).

*Corynebacterium glutamicum* is able to take up GlcN (**Figure 1**) by using its glucose specific PTS PtsG (Arndt and Eikmanns, 2008; Uhde et al., 2013). Efficient growth with GlcN required high levels of the endogenous NagB, e.g., in the absence of the repressor protein NanR (Matano et al., 2016). By contrast, high levels of NagA and NagB were not sufficient to support growth with GlcNAc unless *nagE* from *C. glycinophilum* was expressed heterologously (Matano et al., 2014). Recombinant *C. glutamicum* strains carrying a *nanR* deletion and expressing *nagE* from *C. glycinophilum* produced several value-added products from GlcN or GlcNAc.

Here, *C. glutamicum* strains were developed for utilization of MurNAc as carbon, nitrogen and energy source and for MurNAc-based production of the food amino acid L-glutamate, the feed amino acid L-lysine, L-lysine-derived chemicals as well as the carotenoid lycopene.

## Materials and Methods

### Bacterial Strains and Growth Conditions

The strains and plasmids used in this work are listed in **Table 1**. Pre-cultivation of *C. glutamicum* strains was carried out at 30°C in baffled shake flasks using BHI supplemented with 45.5 g/L D-sorbitol. *E. coli* was grown at 37°C in LB (Lysogeny Broth) medium. Kanamycin (50 μg/mL), chloramphenicol (4.5 and 25 μg/mL for *C. glutamicum* and *E. coli*, respectively) or tetracycline (5 μg/mL) were added, if necessary. To adjust both cultures to growth conditions in the Biolector^®^ system (m2pLabs, Baesweiler), precultures were washed after 24 h with TN-buffer and transferred to CGXII medium (Eggeling and Bott, 2005) with 100 mM GlcN and antibiotics, if necessary. After 24 h, these were transferred to 1 mL cultures in the Biolector^®^ system (1100 rpm) with CGXII medium containing and, if not otherwise stated, 25 mM MurNAc (BACHEM, Bubendorf, Switzerland) as sole carbon source or a combination of 25 mM MurNAc and 25 mM GlcNAc. To trigger glutamate production, penicillin G (10 μM) was added in the main culture. The initial OD_600_ was 1 and gene expression from plasmids pVWEx1 and pEC-XT99A was induced by addition of 25 μM IPTG, if not otherwise stated. Correlation factors for light scattering in the Biolector system, OD_600_ and biomass concentrations were determined.

**Table 1:** Plasmids and strains used in this study.

Strain ID	Characteristics	Reference
***E. coli***		
DH5α	*supE*44 Δl*acU*169(ϕ80*dlacZ*ΔM15) *hsdR*17 *recA*1 *endA*1 *gyrA*96 thi-1 *relA*1	Hanahan, 1983
JW2421-1	F-, Δ(*araD*-*araB*)567, Δ*lacZ*4787(:*rrnB*-3), -, Δ*murQ*757::*kan*, rph-1, Δ(*rhaD*-*rhaB*)568, *hsdR*514; Kan^R^	Baba et al., 2006
JW2421-1 (pCXE50_*murQ*)	JW2421-1 carrying pCXE50_*murQ*	this work
***C. glutamicum***		
Δ*nanR*	ATCC 13032 Δ*nanR*	(Matano et al., 2016)
Δ*nanR PQ*	Δ*nanR* carrying pVWEx1_*murP* and pCXE50_*murQ*	This work
Δ*nanR POQ*	Δ*nanR* carrying pVWEx1_*murP^opt^* and pCXE50_*murQ*	This work
Δ*nanR PCQ*	Δ*nanR* carrying pVWEx1_*murP_crr* and pCXE50_*murQ*	This work
Δ*nanR POCQ*	Δ*nanR* carrying pVWEx1_*murP^opt^_crr* and pCXE50_*murQ*	This work
Δ*nanR PCQnE*	Δ*nanR* carrying pVWEx1_*murP_crr*, pCXE50_*murQ* and pEC-XT99A_*nagE*	This work
DM1729Δ*nanR PCQ*	DM1729Δ*nanR* carrying pVWEx1_*murP_crr* and pCXE50_*murQ*	This work
DM1729Δ*nanR*	ATCC 13032 *pycP*^458S^ *hom*^V 59A^ *lysC*^T311I^ *ΔnanR*, L-lysine overproducing strain	Matano et al., 2014
DM1729Δ*nanR PCQnE*	DM1729Δ*nanR* carrying pVWEx1_*murP_crr*, pCXE50_*murQ* and pEC-XT99A_*nagE*	This work
DM1729Δ*nanR PCQ ldcC*	DM1729Δ*nanR* carrying pVWEx1_*murP_crr*, pCXE50_*murQ* and pEC-XT99A_*ldcC*	This work
DM1729Δ*nanR PCQ LPA*	DM1729Δ*nanR* carrying pVWEx1_*murP_crr*, pCXE50_*murQ* and pECXT99A_*lysDH_S.prom_-proC_C.g_*	This work
Δ*crtYEb* Δ*nanR*	lycopene producing derivative of WT carrying in-frame Δ*crtYEb* Δ*nanR*	Matano et al., 2014
Δ*crtYEb* Δ*nanR PCQ*	Deletion of *crtY* and *crtEb* carrying pVWEx1_*murP_crr*, pCXE50_*murQ*	This work
Δ*crtYEb* Δ*nanR PCQnE*	Δ*crtYEb* Δ*nanR* carrying pVWEx1_*murP_crr*, pCXE50_*murQ* and pEC-XT99A_*nagE*	This work
**Plasmids**		
pVWEx1	*E. coli/C. glutamicum* shuttle vector, Kan^R^	Peters-Wendisch et al., 2001
pEC-XT99A	TetR, *C. glutamicum/E. coli* shuttle vector (Ptrc, *lacI*, pGA1 OriV_C.g_.)	Kirchner and Tauch, 2003
pCXE50_*porB*	Constitutive pEf_tu_ promotor, Cm^R^	Lee, 2014
pVWEx1_*murP*	pVWEx1-derivative for IPTG inducible expression of *murP* from *E. coli*, Kan^R^	This work
pVWEx1_*murP^opt^*	pVWEx1-derivative for IPTG inducible expression of the for *C. glutamicum* codon optimized *murP* from *E. coli*, Kan^R^	This work
pVWEx1_*murP_crr*	pVWEx1-derivative for IPTG inducible expression of *murP* and *crr* from *E. coli*, Kan^R^	This work
pVWEx1_*murP^opt^_crr*	pVWEx1-derivative for IPTG inducible expression of the for *C. glutamicum* codon optimized *murP* and *crr* from *E. coli*, Kan^R^	This work
pCXE50_*murQ*	Cm^R^, expressing *murQ* from *E. coli K12*	This work
pEC-XT99A_*nagE*	Tet^R^, expressing *nagE* from *Corynebacterium glycinophilum* DSM45794	This work
pEC-XT99A_*ldcC*	Tet^R^, expressing *ldcC* from *E. coli K12*	This work
pECXT99A_*lysDH_S.prom_-proC_C.g_*	Tet^R^, expressing *lysDH from Silicibacter pomeroyi* and *proC* from *C. glutamicum*	This work

*Kan^*R*^, kanamycin resistance; Tet^*R*^, tetracycline resistance; Cm^*R*^, chloramphenicol resistance.*

### Construction of Expression Vectors

*Escherichia coli* DH5α was used for cloning. Codon usage of *murP, crr* and *murQ* from *E. coli* for *C. glutamicum* was examined using the graphical codon usage analyzer^1^. The analysis showed, that the codon ATA occurred twice in the sequence of *murP*. This codon is rarely used in *C. glutamicum* and was changed to the more frequently used codon of ATC via site directed mutagenesis (SDM). The mutated variation of *murP* was called *murP^opt^*. The genes of *murP, crr* and *murQ* were amplified via PCR from genomic DNA of *E. coli* K-12, while *nagE* from *C. glycinophilum*, was amplified from pVWEx1_*nagE* (Matano et al., 2016). The primers used in this study (see **Supplementary Table S1**) were obtained from Metabion international AG, Planegg. Using Gibson assembly (Gibson, 2011), the vectors pVWEx1_*murP*, pVWEx1_*murP^opt^*, pVWEx1_*murPcrr*, pVWEx1_*murP^opt^crr*, pCXE50_*murQ* and pEC-XT99A_*nagE* were constructed. The vectors pVWEx1 and pEC-XT99A are IPTG inducible while pCXE50 has a constitutive EF_tu_ promotor. *E. coli* was transformed by the CaCl_2_ method while transformation through electroporation was applied for *C. glutamicum* at 2500 V, 25 μF and 200 Ω.

### Carotenoid Extraction

Lycopene was extracted as described before (Heider et al., 2012). In short, 5 wells each containing 1 ml cell suspension were combined and pelleted in safe lock micro reaction tubes by centrifugation at 10,000 *g* for 15 min and resuspended in 800 μL of a 7:3 methanol/acetone mixture and incubated for 15 min at 60°C and 750 rpm in a thermomixer (Eppendorf). The cell debris was removed by centrifugation and the supernatant used for HPLC analysis. The procedure was repeated to ensure complete extraction until white pellets were obtained.

### Quantitation of Fermentation Products

The quantification of MurNAc, GlcNAc, lycopene, L-glutamate, and L-lysine was conducted by HPLC analysis (1200 series HPLC system, Agilent Technologies Sales & Services GmbH & Co. KG, Waldbronn). The supernatant of 1 ml pelleted cell suspension was diluted and analyzed. For quantification of organic acids, the carbo column (300 × 8 mm, 10 μm particle size, 25 Å pore diameter, CS Chromatographie Service GmbH) and a refractive index detector (RID G1362A, 1200 series, Agilent Technologies) was used for quantification of MurNAc and GlcNAc with 5 mM H_2_SO_4_ as buffer.

Applying OPA derivatisation, L-lysine and L-glutamate were analyzed using an RP8 column with a sodium acetate (0.25 v/v %) buffer at pH 6 and a 1:50 dilution with an internal L-asparagine standard. Using the RP18 column with a Methanol-Milli-Q-water mixture (9:1), lycopene was quantified (Heider et al., 2012).

## Results

### Metabolic Engineering of *C. glutamicum* for Growth With MurNAc as Carbon Source

**FIGURE 2:**
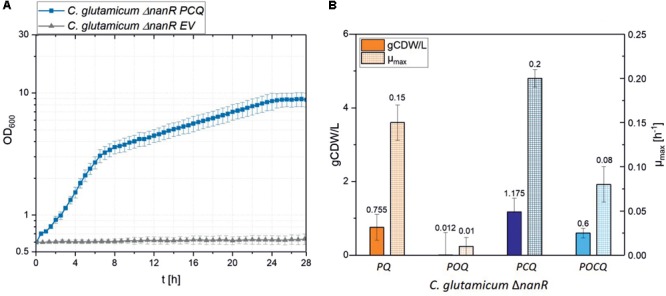
**(A)** Growth parameters of various *C. glutamicum* strains in MurNAc minimal medium. Growth of the strains *C. glutamicum Δ*nanR EV (empty vector) and *C. glutamicum Δ*nanR PCQ was monitored for 25 h at 30°C and 1100 rpm in the Biolector with CGXII medium containing IPTG 25 μM and 20 mM MurNAc. **(B)** Biomass concentrations (gCDW/L; filled) and maximal growth rates (μmax, checkered) of the strains PQ, POQ, PCQ and POCQ grown for 48 h in the Biolector^®^ system in CGXII medium with MurNAc 20 mM. Values and error bars represent means and standard deviations of triplicates.

*Corynebacterium glutamicum*, which has been engineered to utilize GlcN and GlcNAc (Matano et al., 2014, 2016), cannot utilize MurNAc since no growth was observed in minimal medium with 25 mM MurNAc and 25 ± 0.1 mM MurNAc remained in the growth medium after 25 h of incubation (**Figure 2A**). As expected, the *C. glutamicum* genome lacks genes encoding a MurNAc PTS and MurNAc-6-phosphate etherase for uptake and conversion of MurNAc to GlcNAc-6-phosphate, an endogenous intermediate of *C. glutamicum* metabolism. As described in Section “Materials and Methods,” the genes for the MurNAc PTS *murP* from *E. coli* or codon optimized allele *murP*^opt^ (Nuoffer et al., 1988; Tchieu et al., 2001; Dahl et al., 2004) were cloned into the IPTG-inducible plasmid pVWEx1 alone or as operon with *crr*. The gene for the MurNAc-6-phosphate etherase *murQ* from *E. coli* (Jaeger et al., 2005) was cloned into the constitutive expression vector pCXE50 (Lee, 2014). Functional expression of *murQ* from pCXE50*_murQ* was tested by complementation of the *E. coli murQ* mutant *E. coli* JW2421-1. While *E. coli JW2421-1(*pCXE50*_murQ)* utilized MurNAc as sole carbon source (ΔOD_600_ of 3.2 ± 0.1 and μ_max_ of 0.07 ± 0.01 h^-1^), *E. coli JW2421-1ΔmurQ* showed no growth (see **Supplementary Figure S1**). Only *murQ* was tested by complementation of a *E. coli* mutant. We neither tested *murP* nor *crr* because we expected perturbations due to overexpression since MurP is a membrane protein and Crr serves a regulatory function. *C. glutamicum ΔnanR* was transformed with the constructed pVWEx1 plasmids and with the pCXE50_*murQ*. The respective strains were named *C. glutamicum ΔnanR PQ, POQ, PCQ, POCQ* (**Table 1**).

Strains expressing *crr* from *E. coli* grew faster in minimal medium containing 25 mM MurNAc as sole source of carbon and energy than strains lacking *crr* (**Figure 2B**). Strains with native *murP* grew better than strains expressing codon optimized *murP* (**Figure 2B**). IPTG was used at a low concentration (25 μM) to induce heterologous gene expression, since higher concentrations slowed growth (**Table 2**). This is not unexpected and presumably due to too high expression of transport protein genes as seen previously for *dccT* (Youn et al., 2008) and *dctA* (Youn et al., 2009), coding for dicarboxylate transporters. With 25 μM IPTG, strain *ΔnanR PCQ* expressing native *murP, crr* and *murQ* grew in minimal medium containing 25 mM MurNAc to a biomass concentration of 1.2 ± 0.3 gCDW/L and with 50 mM MurNAc to a biomass concentration of 2.0 ± 0.2 gCDW/L (**Table 2**). Biphasic exponential growth was observed: faster growth between 0 and 6 h and slower growth between 6 and 27 h (**Figure 2A**). The curves appear linear as the Y axis has been logarithmized (**Figure 2**). During the transition from the first to the second growth phase the medium contained 3.10 ± 0.12 mM lactate. Thus, lactate released by MurQ from MurNAc-6-phosphate may not have been utilized as fast as GlcNAc-6-phosphate, the other product of the MurQ reaction. In consequence, lactate accumulated in the culture medium in the first exponential growth phase and presumably slowed growth in the second exponential growth phase. Transient accumulation of lactate to growth inhibitory concentrations has been observed during growth of *C. glutamicum* with various carbon sources (Engels et al., 2008).

**Table 2:** Growth characteristics of *C. glutamicum ΔnanR PCQ* on different MurNAc and IPTG concentrations.

C_MurNAc_	C_IPTG_	gCDW/L	μ_max1_	μ_max2_	Y_x/S_
[mM]	[μM]		[h^-1^]	[h^-1^]	[g⋅mol^-1^]
20	25	1.2 ± 0.3	0.25 ± 0.01	0.05 ± 0.01	58.0 ± 0.1
	50	1.0 ± 0.4	0.24 ± 0.01	0.04 ± 0.01	51.4 ± 0.1
	100	1.0 ± 0.1	0.26 ± 0.02	0.05 ± 0.01	54.5 ± 0.1
50	25	2.0 ± 0.2	0.25 ± 0.04	0.04 ± 0.01	38.4 ± 0.1
	50	1.7 ± 0.5	0.26 ± 0.01	0.05 ± 0.01	35.1 ± 0.1
	100	1.5 ± 0.2	0.21 ± 0.02	0.04 ± 0.01	30.4 ± 0.1

*The final biomass concentrations reached [given as g of cell dry weight (CDW) per L], maximal specific growth rates of the first (μ_*max1*_) and second growth phase (μ_*max2*_), and the biomass yield coefficients of cell dry weight formed per used substrate used (Y_*X/S*_) are given as means with standard deviations.*

**FIGURE 3:**
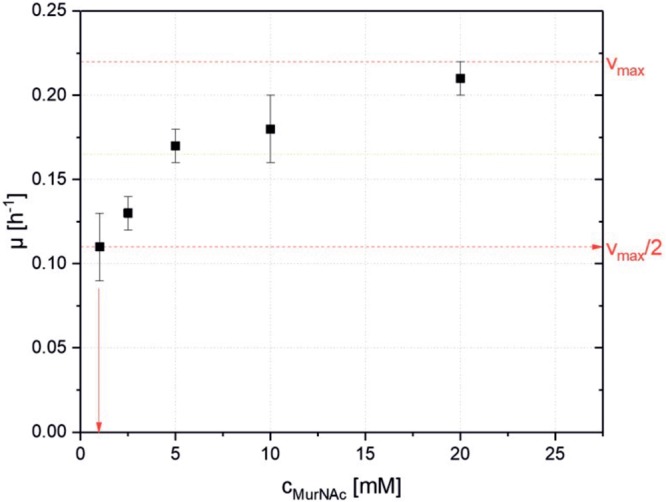
Specific maximal growth rates of *C. glutamicum* ΔnanR PCQ as function of the used MurNAc concentrations. Values and error bars represent means and standard deviations of triplicates.

As PTS systems typically support growth on their cognate substrates with high affinity, the dependence of the growth rate on the initial MurNAc concentration in the growth medium was determined using strain *ΔnanR PCQ*. Different concentrations of MurNAc (1, 2.5, 5, 10, and 20 mM) were used and the maximal growth rates were plotted against the MurNAc concentration to derive the maximal growth rate of 0.22 h^-1^ and the Monod constant of 0.9 ± 0.1 mM as shown in **Figure 3**. A sub-millimolar Monod constant is typical for PTS mediated uptake.

### Comparative Analysis of Growth With MurNAc and/or GlcNAc

**FIGURE 4:**
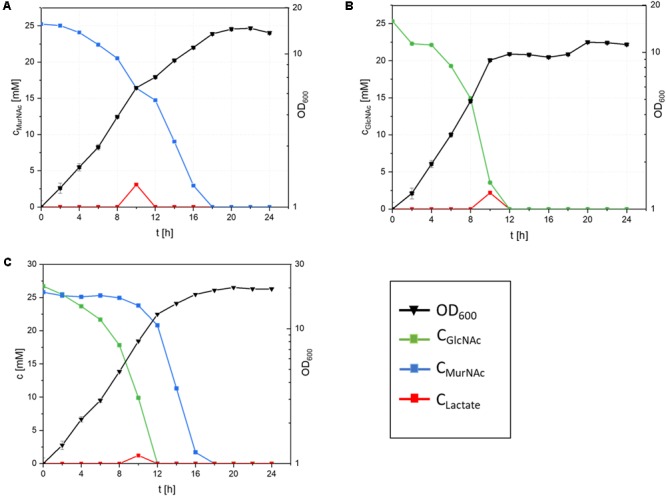
Cultivation of *C. glutamicum* ΔnanR PCQnE in minimal medium with 25 mM MurNAc **(A)**, 25 mM GlcNAc **(B)** or a mixture of 25 mM MurNAc and 25 mM GlcNAc **(C)**. Incubation was done with 25 μM IPTG at 30°C and 130 rpm. OD_600_ (filled triangles), the concentrations of GlcNAc (blue squares), MurNAc (green squares) and lactate (red columns) are given as means and standard deviations of triplicates.

Growth of recombinant *C. glutamicum* with MurNAc and/or GlcNAc as sole carbon sources was compared (**Figure 4** and **Table 3**). With 25 mM MurNAc *C. glutamicum*Δ*nanR PCQnE* grew to a biomass concentration of 3.0 ± 0.1 gCDW/L, while the maximal biomass concentration was only 2.4 ± 0.1 gCDW/L with GlcNAc. The higher biomass concentration observed with MurNAc in comparison to GlcNAc indicated that lactate released from MurNAc by MurQ contributed to biomass formation. However, the biomass yield was higher with GlcNAc (0.44 ± 0.01 g⋅g^-1^) than with MurNAc (0.39 ± 0.02 g⋅g^-1^). GlcNAc catabolism was faster than MurNAc catabolism as the maximal growth rates and the specific substrate uptake rates were lower with MurNAc (0.22 ± 0.10 h^-1^ and 1.80 ± 0.10 mmol⋅g^-1^⋅h^-1^) than with GlcNAc (0.30 ± 0.01 h^-1^ and 3.00 ± 0.10 mmol⋅g^-1^⋅h^-1^) as shown in **Table 3**.

**Table 3:** Cultivation parameters of *C. glutamicum ΔnanR PCQnE* growing on MurNAc (25 mM), GlcNAc (25 mM) or a MurNAc-GlcNAc-mixture (both 25 mM).

	MurNAc	GlcNAc	MurNAc-GlcNAc mix
ΔOD_600_ [-]	12.0 ± 0.1	9.6 ± 0.1	17.7 ± 0.1
ΔS MurNAc [mM]	25.3 ± 0.1	–	25.8 ± 0.1
ΔS GlcNAc[mM]	–	25.3 ± 0.1	26.7 ± 0.1
Y^1^_X/S_ [g⋅mmol^-1^]	0.14 ± 0.01	0.09 ± 0.01	0.11 ± 0.10
μmax^1^ [h^-1^]	0.18 ± 0.01	0.22 ± 0.01	0.18 ± 0.01
qS^1^ [mmol⋅g^-1^⋅h^-1^]	1.27 ± 0.10	2.42 ± 0.10	1.68 ± 0.10
Y^2^_X/S_ [g⋅mol^-1^]	0.11 ± 0.01	–	0.08 ± 0.10
μmax^2^ [h^-1^]	0.10 ± 0.01	–	0.07 ± 0.20
qS^2^ [mmol⋅g^-1^⋅h^-1^]	0.87 ± 0.10	–	0.90 ± 0.20

*The parameters given are ΔOD_*600*_ for biomass formed and ΔS for substrate used. Monophasic growth was observed with GlcNAc. With MurNAc, two phases were observed, and biomass yield coefficients of cell dry weight formed per used substrate used (Y_*X/S*_), maximal specific growth rates (μmax) and specific substrate uptake rates (qS) are given for the phase 1 and phase 2. With MurNAc+GlcNAc, Y_*X/S*_, μmax and qS are reported for the phase where GlcNAc was utilized exclusively (phase 1; 0–8 h) as well as for the phase when MurNAc was utilized exclusively (phase 2; 12–18 h).*

Unlike *E. coli and B. subtilis*, it is typical for *C. glutamicum* to simultaneously co-utilize carbon substrates present in blends (Blombach and Seibold, 2010). Therefore, *C. glutamicum* strain Δ*nanR PCQnE* was constructed by transforming strain Δ*nanR PCQnE* with plasmid pEC-XT99A-*nagE* for expression of the gene for the GlcNAc-specific PTS uptake system to establish whether MurNAc and GlcNAc are co-utilized or utilized sequentially. *C. glutamicum* strain Δ*nanR PCQnE* were grown with 25 mM MurNAc and/or 25 mM GlcNAc (**Figure 4**). With the blend of MurNAc and GlcNAc *C. glutamicum ΔnanR PCQnE* grew to a biomass concentration of 3.8 ± 0.1 gCDW/L, while a biomass concentration of only 2.1 ± 0.1 g/L was reached in the absence of *nagE*. Determination of the residual substrate concentrations revealed sequential utilization of GlcNAc before MurNAc (**Figure 4C**). Thus, unlike many growth substrates MurNAc and GlcNAc were not co-utilized.

### MurNAc-Based Production of Food and Feed Additives and Derived Chemicals

MurNAc was expected not only to support growth of recombinant *C. glutamicum* strains, but also production of value-added compounds. Therefore, MurNAc was tested as sole carbon source or in blends with GlcNAc for production of the amino acids L-lysine and L-glutamate, the diamine 1,5-diaminopentane, the cyclic non-proteinogenic amino acid L-pipecolic acid, and the carotenoid lycopene.

The lycopene accumulating strain *C. glutamicum* Δ*crtYEb* Δ*nanR* (Matano et al., 2014) was transformed with the plasmids pVWEx1_*murP_crr*, pEC-XT99A_*nagE* and pCXE50*_murQ* as described above and the resulting strains were named Δ*crtYEb* Δ*nanR PCQ* and Δ*crtYEb* Δ*nanR PCQnE*. Cells of both strains accumulated lycopene when grown in MurNAc containing minimal medium. Strain Δ*crtYEb* Δ*nanR PCQ* showed a lycopene content of 0.04 mg ± 0.01 (g CDW)^-1^ in MurNAc minimal medium. Growth of *C. glutamicum* Δ*crtYEb* Δ*nanR PCQnE* with a MurNAc/GlcNAc blend led to a lycopene content of 0.10 ± 0.01 mg (g CDW)^-1^.

The L-lysine producing strains *C. glutamicum* DM1729Δ*nanR PCQ* and DM1729Δ*nanR PCQnE* were constructed based on DM1729Δ*nanR* as described above for lycopene accumulating strains. DM1729Δ*nanR PCQ* produced 7 ± 1 mM L-lysine (Y_P/S_ 0.27 ± 0.05 mmol mmol^-1^) and DM1729Δ*nanR PCQnE* produced 11 ± 1 mM L-lysine (Y_P/S_ 0.21 ± 0.10 mmol mmol^-1^) in minimal medium with either 25 mM MurNAc or a combination of 25 mM MurNAc and 25 mM GlcNAc., whereas 7.6 ± 0.3 mM L-lysine (Y_P/S_ 0.30 ± 0.01 mmol mmol^-1^) have been produced from DM1729PCQnE with 25 mM GlcNAc (**Table 4**).

**Table 4:** Parameters describing concentration in mM and production yield (Y_P/S_) of L-lysine,L-PA and 1,5-diaminopentane after 72 h with either 25 mM GlcNAc, either 25 mM MurNAc or 25mM GlcNAc of indicated strains and glutamate production after 48 h from either 25 mM MurNAc either a mixture of GlcNAc and MurNAc, each 25 mM.

Product	Strain ID	C_Substrate_ [mM]	C _product_ [mM]	Y_P/S_ [mmol⋅mmol^-1^]
Lycopene	*ΔcrtYEb ΔnanR PCQ*	25 mM MurNAc	–	0.04 ± 0.01 mg (gCDW)^-1^
	*ΔcrtYEb ΔnanR PCQnE*	25mM GlcNAc +		0.10 ± 0.01 mg (gCDW)^-1^
		25 mM MurNAc		
L-lysine	DM1729*ΔnanR PCQnE*	25 mM GlcNAc	7.6 ± 0.3	0.30 ± 0.10
	DM1729*ΔnanR PCQnE*	25 mM GlcNAc +	10.6 ± 0.6	0.21 ± 0.10
		25 mM MurNAc		
	DM1729*ΔnanR*	25mM MurNAc	6.9 ± 0.6	0.27 ± 0.05
1,5-diaminopentane	DM1729*ΔnanR PCQ ldcC*	25 mM MurNAc	4.3 ± 0.1	0.30 ± 0.10
L-PA	DM1729*ΔnanR PCQ LPA*	25 mM MurNAc	4.2 ± 0.2	0.35 ± 0.10
Glutamate	Δ*nanR PCQ*	25 mM MurNAc	1.0 ± 0.1	0.03 ± 0.00
	Δ*nanR PCQnE*	25mM GlcNAc +	1.9 ± 0.1	0.04 ± 0.00
		25 mM MurNAc		

To test if the L-lysine derived compounds 1,5-diaminopentane and L-pipecolic acid can also be produced from MurNAc, strain DM1729Δ*nanR PCQ* was transformed with either pEC-XT99A-*ldcC* or pEC-XT99A-*lysDH-proC.* 1,5-Diaminopentane can be generated from L-lysine by lysine decarboxylase LdcC and L-pipecolic acid can be generated from L-lysine in a three-step pathway by L-lysine-6-dehydrogenase (encoded by *lysDH* from *S. pomeroyi*), spontaneous ring formation and by pyrroline 5-carboxylate reductase (encoded by endogenous *proC*) (Pérez-García et al., 2016, 2017). Although the strains showed poor growth, *C. glutamicum ΔnanR* DM1729*PCQ ldcC* was able to produce 4.3 ± 0.1 mM of 1,5-diaminopentane (Y_P/S_ 0.30 ± 0.10 mmol mmol^-1^) and *C. glutamicum ΔnanR* DM1729*PCQ LPA* produced 4.0 ± 0.2 mM of L-pipecolic acid (Y_P/S_ 0.35 ± 0.10 mmol mmol^-1^) from MurNAc as sole carbon source as shown in **Table 4**.

L-Glutamate production was accomplished by *C. glutamicum ΔnanR PCQ* and *C. glutamicum ΔnanR PCQnE* using penicillin G as trigger. *C. glutamicum ΔnanR PCQ* accumulated 1 ± 0 mM of L-glutamate from 25 mM MurNAc after 48 h, whereas *C. glutamicum ΔnanR PCQnE* produced 2 ± 0 mM of L-glutamate under these conditions.

## Discussion

In this work, production of food and feed additives by *C. glutamicum* from MurNAc, an alternative carbon source without competing use in human and animal nutrition, has been established. The food amino acid L-glutamate, the feed amino acid L-lysine and the feed additive lycopene were produced from MurNAc, GlcNAc and blends of both hexosamines.

Metabolic engineering of *C. glutamicum* for access to MurNAc relied on *E. coli* genes. Its MurNAc PTS system was active in *C. glutamicum* and strains expressing *crr* in addition were able to grow faster and yield more biomass than the strains without heterologous Crr (**Figure 2**). Crr is a glucose-family specific EIIA component, but it can interact with the PTS-EIIBC components of several members of the glucose PTS family (Barabote and Saier, 2005). *C. glutamicum* has two complete PTS^Glc^ systems (Barabote and Saier, 2005). The finding that MurNAc could be utilized without heterologous Crr indicated that a PTS component of *C. glutamicum* made up for its absence. Moreover, the low Monod constant found for growth of the recombinant with MurNAc (**Figure 3**) indicated that the MurNAc PTS catalyzed high-affinity MurNAc uptake in *C. glutamicum*, although the MurNAc PTS seems to have a lower affinity for its substrate than, e.g., the heterologous expressed GlcNAc specific PTS system NagE from *Corynebacterium glycinophilum* which showed a KM value 3.8 ± 0.6 μM (Ferenci, 1996; Matano et al., 2014). The K_M_ for the etherase MurQ in *E. coli* found in literature had a similar range of value (1.2 mM) (Hadi et al., 2008) with the K_M_ value found experimentally in this study for the MurNAc PTS (0.9 ± 0.1 mM) system, making the two enzymatic steps of up taking, phosphorylation and esterification balanced.

Growth of recombinant *C. glutamicum* with MurNAc was biphasic and lactate accumulated during the interphase (**Figure 4A**). MurNAc differs from GlcNAc only by one additional lactoyl group that is hydrolysed to lactate by etherase MurQ. Although *C. glutamicum* can utilize lactate as sole carbon source (Eikmanns, 2005), lactate accumulated. Utilization of D-lactate requires *dld* encoding quinone-dependent D-lactate dehydrogenase (Stansen et al., 2005; Kato et al., 2010). Utilization of L-lactate requires quinone-dependent L-lactate dehydrogenase which is encoded in the LldR repressed operon *cg3227*-*lldD* (Engels et al., 2008; Georgi et al., 2008). L-Lactate is secreted by *C. glutamicum* under certain conditions, e.g., during growth with glucose when oxygen is limiting, but is quickly re-utilized once *cg3227*-*lldD* is derepressed (Eggeling and Bott, 2005; Stansen et al., 2005; Georgi et al., 2008).

*Corynebacterium glutamicum* co-utilizes glucose simultaneously with many different carbon sources including those that required introduction of heterologous pathways, for example, xylose (requiring xylose isomerase gene from *E. coli* (Kawaguchi et al., 2006), arabinose (requiring the *araBAD* operon from *E. coli* (Kawaguchi et al., 2008; Schneider et al., 2011), cellobiose (requiring β-glucosidase) or glycerol (requiring *E. coli* glycerol kinase and glycerol-3-phosphate dehydrogenase), (Rittmann et al., 2008; Sasaki et al., 2008; Zahoor et al., 2012; Meiswinkel et al., 2013; Wendisch et al., 2016). Only rarely, glucose repression has been observed, for example, during sequential utilization of glucose before ethanol (due to catabolite repression of the alcohol dehydrogenase gene *adhA*) (Gerstmeir et al., 2004) or before glutamate (due to catabolite repression of the operon *gluABCD* encoding the glutamate uptake system) (Wendisch et al., 2000; Blombach and Seibold, 2010). The preferential utilization of GlcNAc before MurNAc by *C. glutamicum ΔnanR PCQnE* may be explained by an offset between fast uptake and hydrolysis of MurNAc yielding GlcNac-6-P and D-lactate followed by fast utilization of GlcNAc-6-P, but accumulation of D-lactate. It is conceivable that overexpression of *dld* would accelerate D-lactate catabolism precluding transient D-lactate accumulation during growth with MurNAc.

A proof-of-concept for MurNAc-based fermentative production of food and feed additives was reached. The engineered strain DM1729Δ*nanR PCQ* and DM1729Δ*nanR PCQnE* showed comparable L-lysine production as observed previously for GlcN and GlcNAc (Matano et al., 2014). Similarly, lycopene production from MurNAc by *C. glutamicum*Δ*crtYEb*Δ*nanR PCQ* was comparable to that observed by similar strains with 100 mM glucose [30 ± 10 μg⋅g (CDW)^-1^] and 100 mM GlcNAc [29.6 ± 4.5 μg⋅g (CDW)^-1^] (Heider et al., 2012; Matano et al., 2014). To establish viable production processes with MurNac as sole or combined carbon source, more work to increase titres, yields and volumetric productivities is needed. Conceptually, however, this work laid the foundation for recycling the cell wall fraction of bacterial biomass from large-scale production processes as substrate for fermentative production of food and feed additives.

## Conclusion

*Corynebacterium glutamicum* was successfully metabolically engineered for utilization of the amino sugar MurNAc as alternative carbon source for growth and production of relevant value-added compounds, specifically L-lysine, L-glutamate and lycopene, from this carbon source lacking competing uses in human and animal nutrition.

## Author Contributions

VW conceived the study. VW and ES planned the experiments. ES and LB performed and analyzed the experiments. ES and LB drafted the manuscript. VW finalized the manuscript. All the authors agreed to the final version of the manuscript.

## Conflict of Interest Statement

The authors declare that the research was conducted in the absence of any commercial or financial relationships that could be construed as a potential conflict of interest.

## Footnotes

1 http://gcua.schoedl.de/

## Abbreviations

μmax: maximal specific growth rate
ΔS: variation of substrate concentration
gCDWL^-1^: gram of Cell Dry Weight per liter
qS: specific substrate uptake rate
Y_P/S_: yield coefficient of cell product per used substrate
Y_P/X_: yield coefficient of product per cell dry weight
Y_X/S_: yield coefficient of cell dry weight per used substrate.
