# Supplementary material for: Production of Food and Feed Additives From Non-food-competing Feedstocks: Valorizing N-acetylmuramic Acid for Amino Acid and Carotenoid Fermentation With Corynebacterium glutamicum
Source: Front Microbiol. 2018 Sep 24;9:2046. doi: 10.3389/fmicb.2018.02046 (PMC6165865; doi:10.3389/fmicb.2018.02046)

Supplementary Data

Production of food and feed additives from non-food-competing feedstocks: Valorizing N-acetylmuramic acid for amino acid and carotenoid fermentation with *Corynebacterium glutamicum*

Elvira Sgobba[#], Luisa Blöbaum[#], Volker F. Wendisch

Chair of Genetics of Prokaryotes, Faculty of Biology & CeBiTec, Bielefeld University, Bielefeld, Germany

# equal contributors

Corresponding author: Volker F. Wendisch, Chair of Genetics of Prokaryotes, Faculty of Biology & CeBiTec, Bielefeld University, Germany, phone: +49-521-106 5611, fax: +49-521-106 5626; volker.wendisch@uni-bielefeld.de

Supplementary Table S1

Supplementary Figure S1

Table S1: Oligonucleotides used in this study

Name	Oligonucleotide 5’-3’	Description	Reference
LB01	*CATGCCTGCAGGTCGACTCTAGAG***GAAAGGAGGCCCTTCAG**ATGGCCAAAGAGATCAGCAG	forward primer for expression of *murP*, overlapping region with pVWEx1, **RBS**	This work
LB02	*GAATTCGAGCTCGGTACCCGGGGATC*TCAGTCCAGATTGACGTTACG	reverse primer for expression of *murP*, overlapping region with pVWEx1	This work
LB21	GAACGTGCATCACCGTGGCAATTAACGTCGCGATCCCCAG	reverse primer for SDM[1] of *murP*	This work
LB22	ATTGCCACGGTGATGCACGTTCCGGCAGATGCTCAGGGAACACTACCCGATG	forward primer for SDM[1] of *murP*	This work
LB23	GACCCCACCAGGCGATCAGGCCGATA	reverse primer for SDM[2] of *murP*	This work
LB24	ATCGCCTGGTGGGGTCTGCCGATGG	forward primer for SDM[2] of *murP*	This work
LB03	*CAGTTTATCGAACAAACCCAT***CTGAAGGGCCTCCTTTC**TCAGTCCAGATTGACGTTACG	reverse primer for expression of *murP*, overlapping region with *crr*, **RBS**	This work
LB04	ATGGGTTTGTTCGATAAACTG	forward primer for expression of *crr*	This work
LB05	*GAATTCGAGCTCGGTACCCGGGGATC*TTACTTCTTGATGCGGATAAC	reverse primer for expression of *crr*, overlapping region with pVWEx1	This work
LB06	GTAATCATCTTTTCAAATTGCAT*TCCTCCTGGACTTCGTGGTGGC*	reverse primer for backbone amplification of pCXE50 with *murQ* overlaps	This work
LB07	GTCAGGTTTTAGACAAGGAATAA*GGCTGTTTTGGCGGATGAGAGAAG*	forward primer for backbone amplification of pCXE50 with *murQ* overlaps	This work
LB08	ATGCAATTTGAAAAGATGATTAC	forward primer for expression of *murQ*	This work
LB09	TTATTCCTTGTCTAAAACCTGACG	reverse primer for expression or *murQ*	This work
LB47	*GAATTCGAGCTCGGTACCCGGG***GAAAGGAGGCCCTTCAG**ATGC	forward primer for expression of *nagE*, pEC-XT99A overlaps, **RBS**	This work
LB48	*GCCTGCAGGTCGACTCTAGAG*CTACTTCTTCTTGGTGACGGTGAAC	reverse primer for expression of *nagE*, overlapping region pEC-XT99A	This work

LB25	CCGACATCATAACGGTTCTG	sequencing primer for *murP*	This work
LB26	GGGATAGCGACGTTAATTGC	sequencing primer for *murP*	This work
LB27	TACCTCGCGTTAATGGACAG	sequencing primer for *murP*	This work
LB28	GAGATCATTGCTCCGCTCTC	sequencing primer for *murP*	This work
LB37	CACGAAGTCCAGGAGGAATG	sequencing primer for *murQ*	This work
LB38	TTCTTCGCGGATGAAAGCAG	sequencing primer for *murQ*	This work
LB44	AGGGCGACACGGAAATGTTG	sequencing primer for pCXE50_*murQ* promotor region	This work
ES44	AATACGCAAACCGCCTCTCC	forward primer for colony PCR with pVWEx1	This work
ES45	TACTTGCCGCCAGGCAAATTC	reverse primer for colony PCR with pVWEx1	This work
ES54	TGTCCACAGGGTAGCTGGTA	forward primer for colony PCR with pCXE50	This work
ES55	ACGGCGTTTCACTTCTGAGT	reverse primer for colony PCR with pCXE50	This work
MM21	GCGCCGACATCATAACGG	forward primer for colony PCR with pVWEx1 or pEC-XT99A	This work
MM22	GGCGTTTCACTTCTGAGTTCGG	reverse primer for colony PCR with pVWEx1 or pEC-XT99A	This work
E237 pEC-*ldcC*F	*CATGGAATTCGAGCTCGGTACCCGGG***GAAAGGAGGCCCTTCAG**ATGAACATCATTGCCATTATGG	Forward primer for PCR *ldcC* from *E. coli*, pEC-XT99A overlaps, **RBS**	This work
E238 pEC-*ldcC*R	CATGCCTGCAGGTCGACTCTAGAGTTATCCCGCCATTTTTAGGACTCG	Reverse primer for PCR *ldcC* from *E. coli*, pEC-XT99A overlaps, **RBS**	This work
E242 pEC-*lysDH*	*CATGGAATTCGAGCTCGGTACCCGGG***GAAAGGAGGCCCTTCAG**ATGCGCTGGAACATTTGTGTC	Forward primer for PCR *lysDH* from *Silicibacter pomeroyi*, pEC-XT99A overlaps, **RBS**	This work
E243 pEC-*proC* R	CATGCCTGCAGGTCGACTCTAGAGCTAGCGCTTTCCGAGTTCTTCAG	Reverse primer for PCR *proC* from *C. glutamicum*, pEC-XT99A overlaps, **RBS**	This work

Italic letters overlapping region, bold letters region ribosomal binding sites + spacer

Figure S1. Growth of *E. coli* JW2421-1 (pCXE50_*murQ) and E. coli* JW2421-1 in CGXII medium with 25 mM MurNAc at 37°C and 1100 rpm. Values and error bars represent means and standard deviations of triplicates.

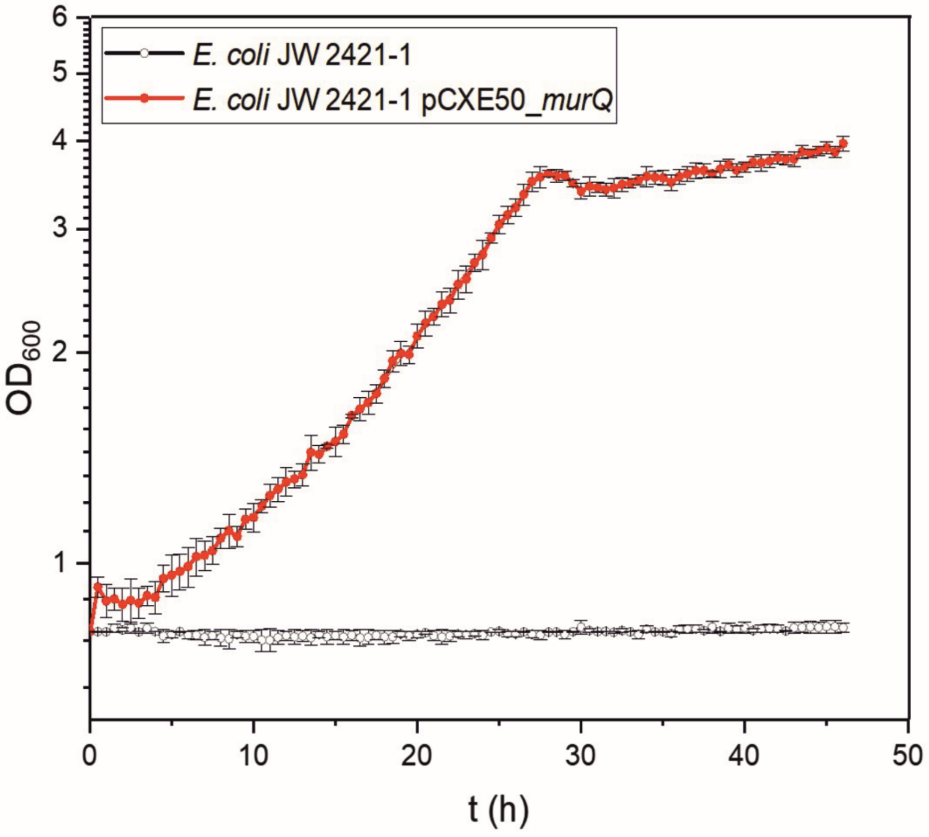

Supplement: Supplementary file 1 [file Data_Sheet_1.pdf]
